# Bone Morphogenetic Protein‐2 Decreases MicroRNA‐30b and MicroRNA‐30c to Promote Vascular Smooth Muscle Cell Calcification

**DOI:** 10.1161/JAHA.112.003905

**Published:** 2012-12-19

**Authors:** Joshua A. F. Balderman, Hae‐Young Lee, Christopher E. Mahoney, Diane E. Handy, Kevin White, Sofia Annis, Djamel Lebeche, Roger J. Hajjar, Joseph Loscalzo, Jane A. Leopold

**Affiliations:** 1 Cardiovascular Division, Department of Medicine, Brigham and Women's Hospital and Harvard Medical School, Boston, MA (J.F.B., H.Y.L., C.E.M., D.E.H., K.W., S.A., J.L., J.A.L.); 2 Cardiovascular Research Center, Mount Sinai School of Medicine, New York, NY (D.L., R.J.H.)

**Keywords:** microRNA, Runx2, smooth muscle cells, vascular calcification

## Abstract

**Background:**

Vascular calcification resembles bone formation and involves vascular smooth muscle cell (SMC) transition to an osteoblast‐like phenotype to express Runx2, a master osteoblast transcription factor. One possible mechanism by which Runx2 protein expression is induced is downregulation of inhibitory microRNAs (miR).

**Methods and Results:**

Human coronary artery SMCs (CASMCs) treated with bone morphogenetic protein‐2 (BMP‐2; 100 ng/mL) demonstrated a 1.7‐fold (*P*<0.02) increase in Runx2 protein expression at 24 hours. A miR microarray and target prediction database analysis independently identified miR‐30b and miR‐30c (miR‐30b‐c) as miRs that regulate Runx2 expression. Real‐time–polymerase chain reaction confirmed that BMP‐2 decreased miR‐30b and miR‐30c expression. A luciferase reporter assay verified that both miR‐30b and miR‐30c bind to the 3′‐untranslated region of Runx2 mRNA to regulate its expression. CASMCs transfected with antagomirs to downregulate miR‐30b‐c demonstrated significantly increased Runx2, intracellular calcium deposition, and mineralization. Conversely, forced expression of miR‐30b‐c by transfection with pre–miR‐30b‐c prevented the increase in Runx2 expression and mineralization of SMCs. Calcified human coronary arteries demonstrated higher levels of BMP‐2 and lower levels of miR‐30b than did noncalcified donor coronary arteries.

**Conclusions:**

BMP‐2 downregulates miR‐30b and miR‐30c to increase Runx2 expression in CASMCs and promote mineralization. Strategies that modulate expression of miR‐30b and miR‐30c may influence vascular calcification.

## Introduction

Vascular calcification is highly prevalent and is associated with a 3‐ to 4‐fold increased risk for cardiovascular morbidity and mortality.^1^ Although once viewed as a consequence of calcium and phosphate imbalance resulting in ectopic mineral deposition, vascular calcification is now viewed as a highly regulated process that recapitulates skeletal bone formation.^2–3^ The central role of vascular smooth muscle cell (SMC) dedifferentiation to an osteoblast‐like phenotype that promotes vascular calcification has been demonstrated.^2–5^ These osteoblast‐like SMCs have decreased expression of smooth muscle cell–specific markers, such as myocardin, smooth muscle myosin heavy chain, and smooth muscle α‐actin; and increased expression of bone‐related proteins, including alkaline phosphatase and osteocalcin.^6^

Runx2 is a master transcription factor of the calcification program that induces differentiation of osteoblasts and chondrocytes.^7–9^ In SMCs, Runx2 is not expressed under basal conditions but is upregulated in response to procalcifying stimuli, including inflammation, oxidant stress, and bone morphogenetic protein‐2 (BMP‐2).^10^ Runx2 is expressed in osteoblast‐like SMCs, and upregulation of Runx2, rather than downregulation of myocardin, drives SMC transition to osteoblast‐like cells.^2–4,11^ The mechanism by which Runx2 protein expression is regulated in SMCs remains incompletely characterized.

MicroRNAs (miRs) are small noncoding sequences of ≈22 nucleotides that bind to the 3′‐untranslated regions (UTRs) of mRNAs to silence gene expression by inhibiting translation or promoting degradation of target mRNAs.^12–15^ miRs are complementary to the target mRNA sequence and only require complete complementarity of a 7‐mer or 8‐mer “seed sequence” for binding to occur. There is evidence to support a role for miRs in vascular calcification as miRs have been implicated in SMC phenotype switching.^5,16–17^ miRs have also been shown to regulate Runx2 expression and osteoblast formation from murine mesenchymal stem cells as well as to regulate osteoblast differentiation.^18–21^ To date, the relationship between miRs and SMC Runx2 expression remains unknown. We, therefore, hypothesized that BMP‐2 downregulates the expression of select miRs that target the 3′‐UTR of Runx2 to induce Runx2 expression and, thereby, SMC calcification.

## Methods

### Cell Culture

Human coronary artery smooth muscle cell (CASMCs; Lonza) were maintained in smooth muscle basal medium (Lonza) supplemented with 5% fetal bovine serum, 0.2% human basic fibroblastic growth factor, 0.1% insulin, and 0.1% human epidermal growth factor without antibiotics at 37°C in 5% CO_2_. For von Kossa and intracellular calcium deposition studies, medium was supplemented with 5 mmol/L β‐glycerophosphate. Cells were passaged at confluence with 0.5% trypsin/EDTA and experiments were performed on cells from passages 3 to 7. In select experiments, cells were stimulated with recombinant human BMP‐2 (R&D Systems; 0 to 200 ng/mL) or vehicle control (4 mmol/L HCl, 0.1% BSA) for up to 14 days.

### von Kossa Staining

Cells were fixed with 10% formalin for 1 hour at 25°C. The cells were then washed with distilled water, 3% silver nitrate solution (Polysciences) was added to each well, and the cells were exposed to UV light for 1 hour. The cells were next washed with distilled water and sodium thiophosphate (5%) was added for 5 minutes. Calcium‐phosphate deposits were detected as black‐stained areas. Images were captured using a VersaDoc (BioRad) scanning system, and densitometry was performed using Adobe Photoshop CS2 software.

### Intracellular Calcium Deposition

Cells were washed with PBS and decalcified with 0.6 mmol/L HCl at 4°C for 24 hours. Calcium released from the cell cultures into the supernatant was determined colorimetrically by the *o*‐cresolphthalein method using the Calcium Colorimetric Assay (BioVision). Calcium content was normalized to total cell protein and expressed as μg/mg cell protein.

### Transfection of miR Antagomirs and Precursors

To inhibit miR expression, cells were transfected with antagomirs (100 pmol) of miR‐30b (anti–miR‐30b; Ambion), miR‐30c (anti–miR‐30c; Ambion), or a combination of both antagomirs (50 pmol), using the siPORT NeoFX transfection reagent (Ambion) according to the manufacturer's instructions. Transfection using a random sequence anti‐miR (Ambion) was used as a negative control. Antagomirs were complexed with the transfection reagent in Opti‐MEM I Reduced Serum Medium (Invitrogen) and added directly to CASMCs in fresh full growth medium. Cells were then cultured for 48 hours before treatment with BMP‐2 (100 ng/mL) or vehicle control.

To force expression of the miRs, cells were transfected with 100 pmol of mature miR‐30b (pre–miR‐30b; Ambion), mature miR‐30c (pre–miR‐30c; Ambion), or a combination of both pre‐miRs (50 pmol), using the siPORT NeoFX transfection reagent (Ambion) according to the manufacturer's instructions. A random sequence pre‐miR (Ambion) was used as a negative control. Cells were treated as described for the transfection of antagomirs.

### *Smad1* siRNA Transfection

To decrease *Smad1* expression, CASMCs were transfected with *Smad*1 Stealth Select RNAI siRNA (5′‐GGAACUGCAACUACCAUCAUGGAUU‐3′) or Stealth RNAI siRNA Negative Control LO GC using Lipofectamine 2000 (Invitrogen) for 5 hours in OptiMEM I medium. After this time, cells were placed in full growth medium and experiments were performed after 48 hours.

### mRNA and miR Isolation and Quantitative Real‐Time–Polymerase Chain Reaction

Total RNA containing miRs was prepared from cells using the RNeasy RNA isolation kit (Qiagen) according to the manufacturer's instructions. For the real‐time–polymerase chain reactions (RT‐PCRs), 1 μg of RNA was used to generate cDNA with oligo(dT) primers using the SuperScript III First‐Strand Synthesis System for RT‐PCR (Invitrogen). Quantitative RT‐PCR was performed using 1 ng of first‐strand cDNA, TaqMan Universal Master Mix, and 20X TaqMan Runx2 or GAPDH Gene Expression Assays (Applied Biosystems). Samples were run on an Applied Biosystems 7900 HT Fast RT‐PCR system. Quantitation of data was performed using the comparative *C*_T_ (∆∆*C*_T_) method using GAPDH gene expression as an endogenous reference.

Mature miRNA expression levels were also analyzed by quantitative RT‐PCR using TaqMan miR assays (Applied Biosystems) for human miR‐30a, miR‐30b, miR‐30c, miR‐30d, or miR‐30e (Applied Biosystems) using the TaqMan miR reverse transcription kit (Applied Biosystems) according to the manufacturer's instructions. Real‐time PCR was performed on the resulting complementary DNA using miR‐30a, miR‐30b, miR‐30c, miR‐30d, miR‐30e, or RNU48 TaqMan miRNA Gene Expression Assays and TaqMan Universal PCR Master Mix. Samples were run on in a 7900 HT Fast Real‐Time PCR system (Applied Biosystems) in 9600 Emulation mode. Quantitation of data was performed using the comparative *C*_T_ (∆∆*C*_T_) method using RNU48 expression as the endogenous reference.

### miR Microarray

miR microarray was performed using the Human MiRNA Genome V2.0 Complete RT^2^ PCR Array, which includes 752 expressed human miRs, according to the manufacturer's instructions (SABiosciences). Data were analyzed using software provided by the manufacturer and findings were confirmed in a duplicate experiment.

### Luciferase Reporter Assay

COS7 cells (ATCC) were grown to 50% confluence in 6‐well plates in high‐glucose Dulbecco's Modified Eagle's Medium (Invitrogen) supplemented with 10% fetal bovine serum. Cells were transfected with 1000 ng of a reporter plasmid containing the 3′‐UTR sequence of Runx2 (NM_001015051) that was cloned downstream of the firefly luciferase gene using Lipofectamine 2000. Cells were cotransfected with 10 nmol/L pre–miR‐30b, pre–miR‐30c, both pre‐miRs (5 nmol/L each), or a negative control (Ambion). After 48 hours, firefly luciferase was measured in cell lysates using the britelite plus Reporter Gene Assay System (PerkinElmer) and a 20/20^n^ Turner BioSystems Luminometer.

### Western Immunoblotting and Densitometry

Cells were harvested and centrifuged at 1000*g* at 4°C for 5 minutes, after which the supernatant was discarded and the samples were frozen at −80°C overnight. The pellet was homogenized and 20 to 50 μg cell protein was added per lane. Proteins were size‐fractionated electrophoretically using SDS‐PAGE and transferred to polyvinylidene fluoride membranes. After blocking with 5% skim milk, the polyvinylidene fluoride membranes were incubated with antibodies to smooth muscle myosin heavy chain, smooth muscle α‐actin, desmin, tubulin, Runx2, actin, osteopontin, or osteocalcin (Abcam), and visualized using the enhanced chemiluminescent system (Amersham Biosciences). Densitometry was performed on a minimum of 3 immunoblots using a VersaDoc (BioRad) scanning system to quantify band density.

### Alkaline Phosphatase Activity

Alkaline phosphatase activity was assayed using 5‐bromo‐4‐chloro‐3′‐indolyphosphate (BCIP) and nitro‐blue tetrazolium (NBT), which in combination yield an intense black‐purple color when reacted with alkaline phosphatase. Briefly, cells were washed twice with phosphate buffered saline, fixed with 10% formalin for 1 hour at 25°C, and washed in water. Samples were then incubated with BCIP/NBT (Promega) for 30 minutes and processed according to the manufacturer's instructions. Images of 3 random ×10 fields were captured using a Nikon TMS microscope equipped with a Nikon Coolpix 4300 camera. Colorimetric detection of alkaline phosphatase activity was quantified using Adobe Photoshop CS2 software.

### Human Coronary Artery Specimens

Human coronary artery sections were isolated at the time of explant, immediately snap frozen in liquid nitrogen, and maintained in a tissue bank with institutional review board approval from Partners Human Research Committee and Mount Sinai School of Medicine. Calcification was detected by incubation with Alizarin Red (American Mastertech Scientific) for 5 minutes according to manufacturer's instructions. For immunohistochemistry, 2‐mm sections were fixed in formalin for 24 hours and embedded in paraffin, and 5‐μm sections were immunostained for detection of BMP‐2 (Abcam). In situ hybridization for detection of miR‐30b was performed by treating deparaffinized and rehydrated sections with proteinase K (10 μg/mL) for 15 minutes at 25°C. Samples were incubated with hybridization buffer (Sigma) for 1 hour followed by overnight incubation with 10 nmol/L miR‐30b or scramble miRCURY LNA Detection probes, FAM‐labeled (EXIQON) at 55°C. Immunodetection was performed using an anti‐FITC horseradish peroxidase–conjugated antibody (1:100; DAKO) followed by tyramide (PerkinElmer) amplification. Sections were then incubated with NeutrAvidin‐conjugated alkaline phosphatase (1:200; Thermo Scientific) in maleate buffer and NBT/BCIP (Roche) to visualize miR‐30b. Slides were left at room temperature for 24 hours, and dark purple areas were considered to be positive staining. Images were captured at ×100 using an Olympus BX51 microscope equipped with PictureTaker software.

### Statistical Analysis

All experiments were performed a minimum of 3 times in duplicate. The Shapiro–Wilk test was used to test normality. Data are presented as the mean±SEM, and ≥2 independent groups were compared using an unpaired Student *t* test or 1‐way ANOVA, respectively, followed by a post hoc Tukey test. A repeated‐ measures ANOVA was used for comparisons over time. All analyses were performed using Origin 8.5 software (OriginLab). A *P* value of <0.05 was considered significant.

## Results

### BMP‐2 Promotes Calcification of CASMCs by Increasing Runx2 Expression

**Figure 1.**
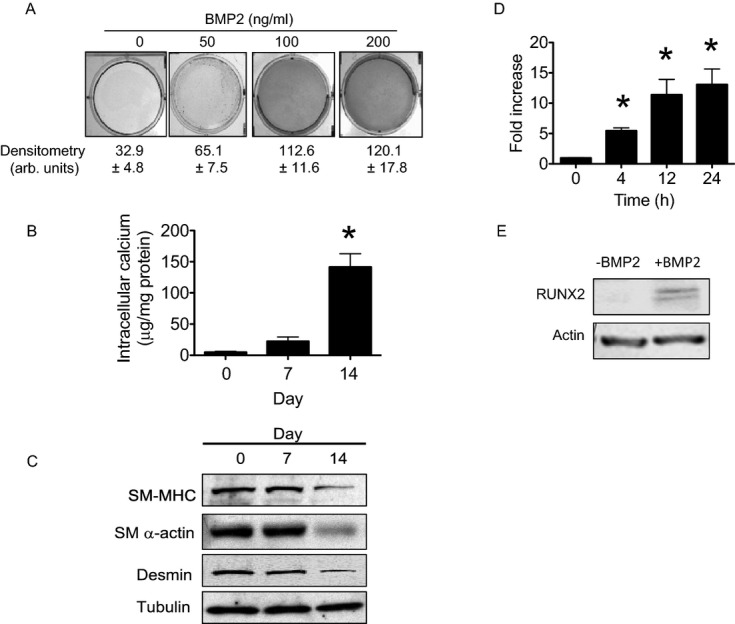
BMP‐2 increases Runx2 expression and calcification of human coronary artery smooth muscle cells (CASMCs). A, Human CASMCs were treated with BMP‐2 (0, 50, 100 or 200 ng/mL) for 14 days and calcification was assessed by von Kossa staining. Representative images are shown (n=4). B, Cellular calcium levels were assessed in CASMCs treated with BMP‐2 (100 ng/mL) for up to 14 days. *P<0.01 vs day 0 (n=4). C, CASMC transition to a calcifying phenotype was confirmed by examining expression of smooth muscle myosin heavy chain (SM‐MHC), smooth muscle α‐actin (SM α‐actin), and desmin. Tubulin expression levels were examined as loading controls and representative blots are shown (n=3). D, Runx2 mRNA expression was measured by quantitative real‐time–polymerase chain reaction in CASMCs treated with BMP‐2 (100 ng/mL) for up to 24 hours (n=6) and E, corresponding protein levels were evaluated at 24 hours. Representative blots are shown (n=4). *P<0.01 vs 0 hour. BMP‐2 indicates bone morphogenetic protein‐2.

To establish the dose–response and time course of BMP‐2–mediated calcification of CASMCs, we treated cells with increasing concentrations of BMP‐2 and examined calcification by von Kossa staining after 14 days. In CASMCs treated with BMP‐2 (0, 50, 100, or 200 ng/mL), there was a dose‐dependent increase in calcification with a maximal effect observed in cells treated with BMP‐2 (100 ng/mL; Figure 1A). We therefore elected to perform further studies in cells treated with this dose. Mineralization was associated with an increase in intracellular Ca^2+^ deposition in BMP‐2–treated CASMCs after 7 days (5.2±1.2 μg/mg vs 62.4±4.9 μg/mg protein, *P*<0.01) that was elevated further after 14 days (144.7±21.3 μg/mg protein, *P*<0.01; Figure 1B). Consistent with CASMC transition to an osteoblast‐like phenotype, there was a decrease in the expression of the smooth muscle–specific markers, smooth muscle myosin heavy chain, smooth muscle α‐actin, and desmin that was apparent after 14 days (Figure 1C). Thus, under these experimental conditions, BMP‐2 increased CASMC calcification in vitro.

### BMP‐2 Increases Runx2 Expression by Downregulating miR‐30b‐c

As Runx2 expression is necessary for vascular SMC transition to a calcifying phenotype,^4,11^ we examined Runx2 mRNA and protein expression in BMP‐2–stimulated cells. BMP‐2 increased Runx2 mRNA levels by 5.5‐fold at 4 hours and a 13.1‐fold by 24 hours compared with vehicle‐treated cells (*P*<0.01; Figure 1D). While Runx2 protein was not expressed in vehicle‐treated cells, in BMP‐2–treated cells Runx2 protein expression was evident at 24 hours (1.7±0.4‐fold, *P*<0.02; Figure 1E).

**Figure 2.**
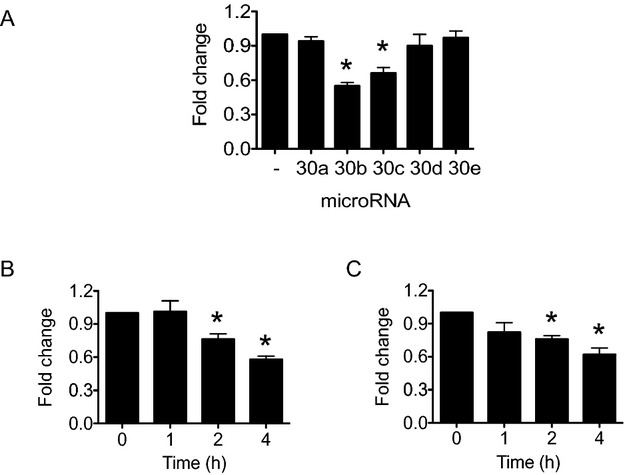
BMP‐2 decreases miR‐30b and miR‐30c expression. Human CASMCs were treated with BMP‐2 (100 ng/mL) for 4 hours and (A) expression of miR30 family members (miR‐30a, miR‐30b, miR‐30c, miR‐30d, and miR‐30e) was evaluated by quantitative real‐time–polymerase chain reaction ( PCR). *P<0.01 vs untreated, (n=4). (B, C) To determine the temporal relationship between changes in Runx2 and miR‐30b and miR‐30c expression, CASMCs were treated with BMP‐2 (100 ng/mL) for up to 4 hours and miR‐30b and miR‐30c expression was measured by quantitative real‐time PCR. *P<0.01 vs 0 hour (n=4). BMP‐2 indicates bone morphogenetic protein‐2; CASMC, coronary artery smooth muscle cell; miR, microRNA.

Next, to determine if BMP‐2 increased Runx2 expression by downregulating the expression of inhibitory miRs, we treated CASMCs with BMP‐2 for 4 hours and performed an miR microarray to identify candidates that were downregulated by BMP‐2. Of 11 miRs predicted to target the 3′‐UTR of human Runx2 (miRbase, TargetScan),^22–25^ the microarray revealed that 7 miRs were poorly expressed in CASMCs (miR‐133, miR‐205, miR‐338, miR‐135, miR‐203, miR‐202, and miR‐207) and that BMP‐2 did not significantly change the expression of 3 miRs (miR‐23, miR‐218, and miR‐34); however, BMP‐2 decreased the expression of miR‐30b by 6.2‐fold (*P*<0.01) and miR‐30c by 5.5‐fold (*P*<0.01) compared with untreated CASMCs. Given that members of the miR‐30 family (miR‐30a‐e) share a similar seed sequence, we confirmed the microarray results using qRT‐PCR to assess changes in miR‐30a‐e expression. In CASMCs treated with BMP‐2 for up to 4 hours, there was a significant decrease in miR‐30b (43%, *P*<0.01) and miR‐30c (34%, *P*<0.01) expression; however, there was no decrease in miR30a, miR30d, or miR30e expression levels (Figure 2A). Expression of miR30b (Figure 2B) and miR30c (Figure 2C) was dowregulated significantly by BMP‐2 after 2 hours, before the observed upregulation of Runx2 mRNA, indicating that these miRs are plausible candidates to regulate Runx2 expression.

**Figure 3.**
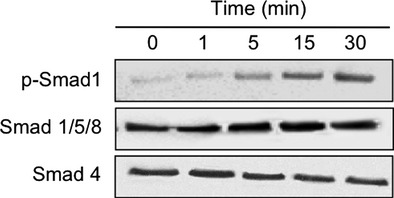
BMP‐2 increases Smad 1/5/8 activation. Human coronary artery smooth muscle cells were treated with BMP‐2 and phosphorylation of Smad 1/5/8 (p‐Smad 1/5/8) was examined up to 30 minutes. Total levels of Smad 1/5/8 and of the regulatory Smad, Smad 4, were also examined. Representative blots are shown (n=3). BMP‐2 indicates bone morphogenetic protein‐2.

**Figure 4.**
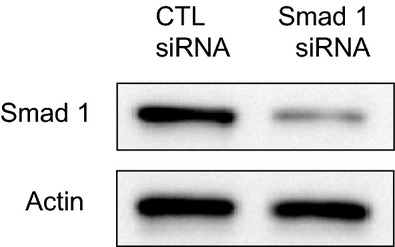
*Smad1* siRNA transfection. Human coronary artery smooth muscle cells were transfected with *Smad1* siRNA or a scrambled control siRNA (CTL siRNA) and *Smad1* expression was examined after 24 hours. A representative blot is shown (n=3).

BMP‐2 is also known to activate Smad signaling, and Smads have been implicated in the regulation of miR expression, albeit by increasing miR maturation.^26^ Therefore, we next examined Smad 1/5/8 activation in BMP‐2–stimulated CASMCs (Figure 3). Here, we found that BMP‐2 increased phosphorylation of Smad 1/5/8 with no change in Smad 4 expression. To confirm that downregulation of miR‐30b and miR‐30c occurred through an Smad‐independent pathway, we transfected CASMCs with an siRNA to decrease *Smad1* mRNA levels by 87% and protein expression by 68%, or a control siRNA (Figure 4), and incubated cells with BMP‐2 for 4 hours. In control‐transfected cells, BMP‐2 decreased miR‐30b expression by 58.5±3.4% (*P*<0.01), and downregulation of *Smad1* had no further effect (64.6±3.9%, *P*=0.2). Similarly, BMP‐2 downregulated expression of miR‐30c by 63.4±2.9% (*P*<0.01) in control‐transfected cells, with no further decrease observed in *Smad1* siRNA‐transfected cells (66.5±2.0%, *P*=0.2).

### miR‐30b‐c Modulates Runx2 Protein Expression

**Figure 5.**
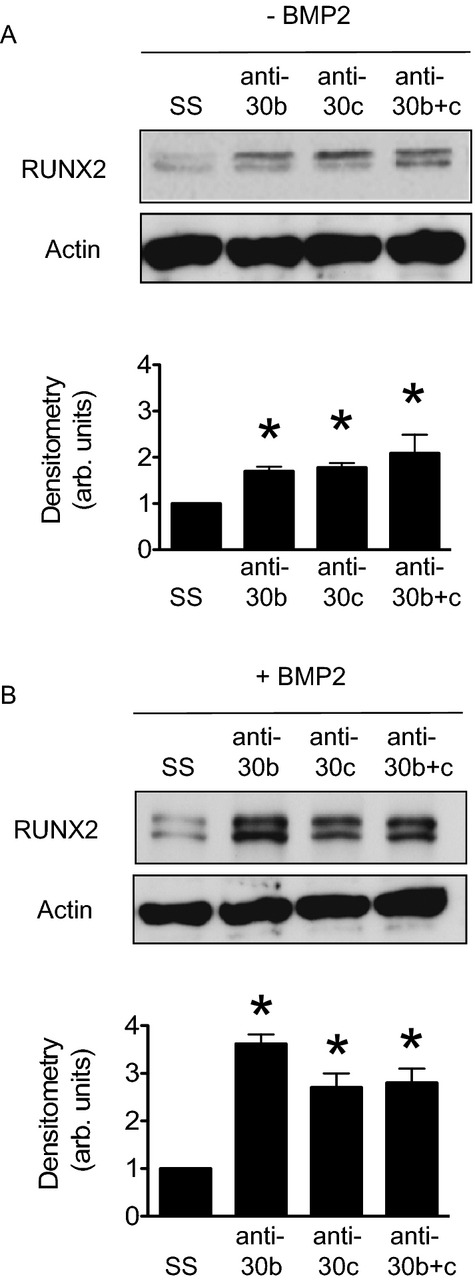
miR‐30b or miR‐30c knockdown increases Runx2 protein expression. Human CASMCs were transfected with an antagomir to miR‐30b (anti–miR‐30b), to miR‐30c (anti–miR‐30c), or to both, or with a scrambled miR sequence (SS) as a control. Runx2 protein levels were determined (A) under basal conditions after 24 hours or (B) following stimulation with BMP‐2 (100 ng/mL) for 24 hours. Densitometry was performed on a minimum of 3 independent blots. Representative blots are shown. *P<0.01 vs SS. BMP‐2 indicates bone morphogenetic protein‐2; CASMC, coronary artery smooth muscle cell; miR, microRNA.

To demonstrate that miR‐30b and miR‐30c play a role in modulating Runx2 expression, we first transfected CASMCs with antagomirs to miR‐30b, miR‐30c, or both, to inhibit their expression by 98%, 95%, or 98%, respectively, compared with cells transfected with a scrambled sequence control miRNA. Compared with control‐transfected cells, downregulation of miR‐30b or miR‐30c, either alone or in combination, resulted in an increase in Runx2 protein expression in the absence of BMP‐2‐stimulation (Figure 5A). In cells that were treated with BMP‐2 for 24 hours, inhibition of miR‐30b or miR‐30c, alone or in combination, resulted in an increase in Runx2 expression that was greater than that observed with BMP‐2 stimulation alone (Figure 5B).

**Figure 6.**
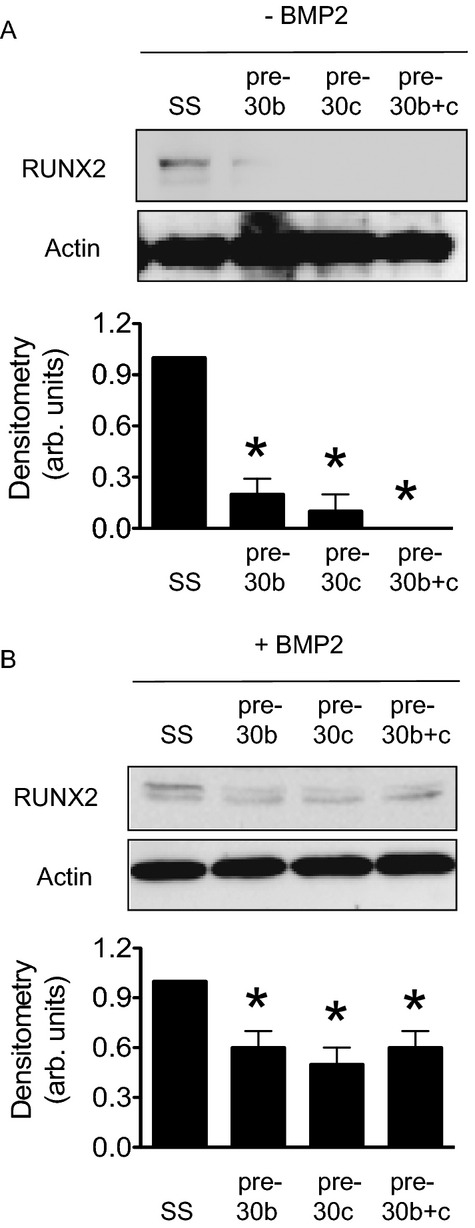
miR‐30b‐c forced expression decreases Runx2 protein expression. Human CASMCs were transfected with pre–miR‐30b, pre–miR‐30c, or both, or with a scrambled miR sequence (SS) as a control. Runx2 protein levels were evaluated (A) under basal conditions after 24 hours or (B) following stimulation with BMP‐2 (100 ng/mL) for 24 hours. Densitometry was performed on a minimum of 3 independent blots. Representative blots are shown. *P<0.01 vs SS. BMP‐2 indicates bone morphogenetic protein‐2; CASMC, coronary artery smooth muscle cell; miR, microRNA.

To confirm these findings, we next transfected cells with mature miR‐30b, mature miR‐30c, or both, to force expression of these miRs to levels 4.8‐fold, 4.5‐fold, and 4.6‐fold, respectively, compared with CASMCs transfected with a scrambled control mature miR. In CASMCs, forced expression of miR‐30b or miR‐30c, alone or in combination, decreased or prevented the induction of Runx2 (Figure 6A). When CASMCs were stimulated with BMP‐2 for 24 hours, forced expression of miR‐30b or miR‐30c limited Runx2 protein expression (Figure 6B). Taken together, these findings indicate that miR‐30b and miR‐30c are endogenous regulators of Runx2 protein expression.

### miR‐30b‐c Target Runx2 Through a Site in the 3′‐UTR

**Figure 7.**
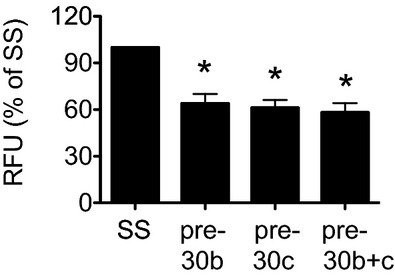
miR‐30b‐c targets Runx2 through a site at the 3′ ‐UTR. A reporter plasmid construct containing the Runx2 3′ ‐UTR and pre–miR‐30b, pre–miR‐30c, or both, were co‐transfected into COS7 cells. A scrambled miRNA sequence (SS) served as the negative control. In this assay, decreased luminosity indicates miRNA binding to the 3′ ‐UTR. Relative fluorescence units (RFUs) were compared against SS. *P<0.04 vs SS. miR indicates microRNA.

Review of the human Runx2 3′‐UTR revealed that there were conserved binding sites for miR‐30b‐c. To demonstrate that miR‐30b and miR‐30c bind to the Runx2 3′‐UTR directly as a mechanism by which to modulate Runx2 expression, we cotransfected COS7 cells with a luciferase reporter plasmid containing the Runx2 3′‐UTR with mature miR‐30b, miR‐30c, both, or a scrambled sequence negative control miR. In this assay, binding of miR‐30b or miR‐30c to the 3′‐UTR inhibits luciferase expression and luminosity is diminished. Compared with scrambled control‐transfected cells, we found that forced expression of miR‐30b, miR‐30c or both decreased luminosity significantly (100.0±1.0% vs 64.1±6.2% vs 61.2±5.2% vs 58.1±6.7%, *P*<0.04), indicating that miR‐30b‐c bind to the 3′‐UTR of Runx2 to inhibit translation (Figure 7).

### miR‐30b‐c Knockdown Increases Expression of Runx2 Target Genes and CASMC Calcification

**Figure 8.**
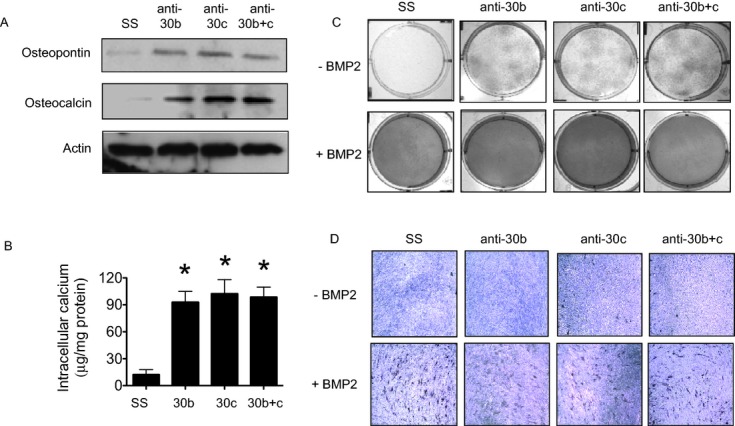
miR‐30b‐c knockdown increases expression of Runx2 target proteins and calcification of human coronary artery smooth muscle cells. Human CASMC were transfected with an antagomir to miR‐30b (anti‐miR‐30b), to miR‐30c (anti‐miR‐30c), to both, or with a scrambled miRNA sequence (SS). After 14 days (A) expression of the Runx2 target proteins osteopontin and osteocalcin was examined. Actin expression levels were examined as a loading control and representative blots are shown (n=3). B, Calcium levels were determined (*P<0.01 vs SS, n=4); and, in the presence or absence of BMP‐2 (100 ng/mL) for the 14‐day treatment period, (C) calcification was evaluated by von Kossa staining (n=3) and (D) activity of the Runx2‐independent protein alkaline phosphatase was assessed using BCIP/NCP staining at 10× magnification (n=4). *P<0.01 vs SS. BMP‐2 indicates bone morphogenetic protein‐2; CASMC, coronary artery smooth muscle cell; BCIP, 5‐bromo‐4‐chloro‐3′‐indolyphosphate; miR, microRNA; NBT, nitro‐blue tetrazolium.

To evaluate the functional consequences of modulating miR‐30b and miR‐30c expression, and thereby Runx2, we first transfected CASMCs with antagomirs to miR‐30b or miR‐30c for 14 days and examined the expression of the Runx2 target genes osteopontin and osteocalcin. In the absence of exogenous BMP‐2, there was an increase in the expression of both osteopontin and osteocalcin (Figure 8A). We next examined intracellular Ca^2+^ deposition in CASMCs transfected with antagomirs to miR‐30b and miR‐30c. Compared with scrambled control‐transfected cells, even in the absence of BMP‐2 stimulation, downregulation of miR‐30b, miR‐30c, or both increased Ca^2+^ deposition (Figure 8B). We also examined calcification by von Kossa staining after 14 days. Here, we found that downregulation of the expression of miR‐30b, miR‐30c, or both resulted in an increase in calcification compared with scrambled control transfected cells (1.0±0.1 vs 3.2±0.2 vs 2.8±0.2 vs 2.5±0.5‐fold, *P*<0.01; Figure 8C); however, this increase was abrogated compared with what was observed in BMP‐2–simulated scrambled control‐transfected CASMCs (Figure 8C).

**Figure 9.**
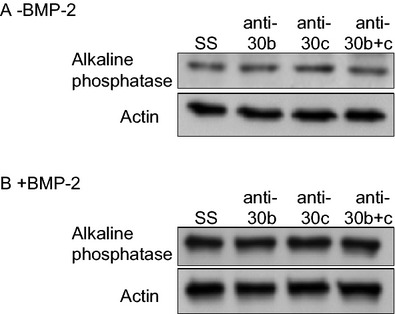
Alkaline phosphatase expression. Human coronary artery smooth muscle cells were transfected with a scrambled control antagomir (SS), antagomirs to miR‐30b, miR‐30c, or both and alkaline phosphatase expression was examined after 14 days in (A) the absence of BMP‐2 or (B) in cells treated with BMP‐2. Representative blots are shown (n=3). BMP‐2 indicates bone morphogenetic protein‐2.

To explain the observed difference in the degree of calcification by von Kossa staining between CASMCs with decreased expression of miR‐30b or miR‐30c and BMP‐2–stimulated CASMCs, we examined the expression and activity of alkaline phosphatase, a Runx2‐independent calcification protein that is necessary for mineralization. Compared with scrambled control transfected CASMCs, in cells transfected with antagomirs to miR‐30b, miR‐30c, or both, there was no observed increase in alkaline phosphatase expression (Figure 9A) or activity (1.0±0.0 vs 1.2±0.2 vs 1.1±0.1 vs 1.0±0.0‐fold, *P*=0.93). In contrast, in BMP‐2–stimulated CASMCs, there was an increase in alkaline phosphatase expression (Figure 9B) and activity (2.6±0.2‐fold, *P*<0.01) that was not augmented further by downregulation of miR‐30b (2.1±0.1‐fold), miR‐30c (1.9±0.2‐fold), or both (1.9±0.2‐fold; Figure 8D). Similarly, compared with scrambled control‐transfected CASMCs, forced expression of miR‐30b, miR‐30c, or both had no effect on alkaline phosphatase activity at baseline (1.0±0.0 vs 1.1±0.1 vs 0.9±0.1 vs 1.0±0.1‐fold, *P*=0.96) or following treatment with BMP‐2 (2.2±0.1 vs 2.0±0.1 vs 2.4±0.2‐fold, *P*<0.01 vs BMP‐2).

### miR‐30b Is Downregulated in Human Coronary Artery Atherosclerosis

**Figure 10.**
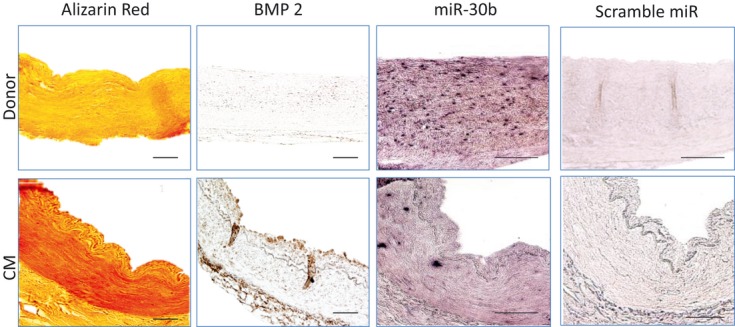
miR‐30b is downregulated in human coronary artery atherosclerosis. Human coronary arteries were isolated at the time of explant from donors without atherosclerotic coronary artery disease (n=3) and individuals with atherosclerotic coronary disease and cardiomyopathy (n=3). Calcification was assessed using Alizarin Red. BMP‐2 expression was determined by immunohistochemistry and miR‐30b expression was examined by in situ hybridization. Representative images are shown. BPM‐2 indicates bone morphogenetic protein‐2; CM, cardiomyopathy; miR, microRNA.

To demonstrate the in vivo relevance of our findings in CASMCs, we next examined human coronary arteries obtained at the time of explant for evidence of calcification. Compared with nonatherosclerotic donor human coronary arteries (n=3), atherosclerotic arteries from patients with cardiomyopathy (n=3) had evidence of vascular calcification by Alizarin Red staining (Figure 10). In calcified atherosclerotic vessels, there was an associated increase in BMP‐2 expression and a concomitant decrease in miR‐30b expression as detected by in situ hybridization compared with the donor vessels (Figure 10).

## Discussion

In our study, we found that BMP‐2 downregulates miR‐30b and miR‐30c to increase Runx2 protein expression and promote CASMC calcification. BMP‐2 downregulates miR‐30b and miR‐30c after 2 hours, a time point before the observed increase in Runx2 expression. Using a luciferase reporter assay, we also demonstrated that miR‐30b and miR‐30c bind to the Runx2 3′‐UTR to repress Runx2 mRNA translation. By transfecting CASMCs with antagomirs to miR‐30b and miR‐30c, in the absence of BMP‐2 stimulation, we demonstrated that downregulation of these miRs is sufficient to increase Runx2 expression. This, in turn, resulted in increased expression of the Runx2‐dependent genes osteopontin and osteocalcin, increased intracellular Ca^2+^ deposition, and CASMC calcification. Interestingly, CASMC calcification following downregulation of miR‐30b or miR30c alone was less than that observed in BMP‐2–stimulated cells, likely owing to the observation that downregulation of miR‐30b or miR‐30c alone did not increase alkaline phosphatase activity, a finding that has been reported by other investigators.^20^ Nonetheless, basal levels of alkaline phosphatase activity were adequate to promote intracellular Ca^2+^ deposition consistent with CASMC calcification. Moreover, we demonstrated that these findings have relevance for atherosclerotic coronary artery disease by confirming a decrease in miR‐30b expression in human coronary arteries that was associated with increased BMP‐2 expression and vascular calcification.

That BMP‐2 downregulates miRs, in particular miR‐30b and miR‐30c, to increase Runx2 expression in CASMCs is consistent with work in other cell types directly related to bone formation. For example, BMP‐2, a member of the transforming growth factor‐β superfamily, stimulates osteoblastic differentiation in vitro and increases the expression of genes encoding osteoblast phenotype‐related proteins.^21,27^ Findings from several studies provide evidence to demonstrate that BMP‐2 modulates miR expression to increase the expression of osteoblast phenotype‐related proteins and initiate osteogenesis.^19^ In C2C12 cells, BMP‐2 promotes osteogenic differentiation by decreasing miR‐133 expression to increase Runx2 as well as downregulating miR‐206 expression to limit the expression of myogenic differentiation marker proteins.^19,28–30^ In preosteoblast MC3T3‐E1 cells, BMP‐2 decreased miR‐208 to regulate Ets‐1 and osteogenic differentiation.^31^ In contrast, BMP‐2 was also shown to regulate Runx2 expression by upregulating miR‐3960 expression. This, in turn, resulted in decreased expression of homeobox A2, a repressor of Runx2 expression.^32^ In our study, we did not examine the role of the aforementioned miRs in modulating Runx2 expression, as they were not identified by the miR microarray as likely candidates in SMCs; however, this does not exclude the possibility that they may be important for SMC calcification. Moreover, results from these studies may not be broadly applicable to vascular calcification as they were conducted using different primary cells, or cell lines, and culture conditions, and different durations of BMP‐2 exposure, and each focused on a different candidate miR. It remains more likely that in SMCs, BMP‐2 downregulates the expression of a panel of miRs that affect several targets to promote osteogenic differentiation and calcification.

Microarray and bioinformatic analyses identified miR‐30b and miR‐30c as key regulators of Runx2 expression in BMP‐2–stimulated CASMCs. Importantly, while the Runx2 3′‐UTR contains >1 site that is complementary to the seed sequence shared by the miR‐30 family, we found no difference in Runx2 expression when we combined miR‐30b and miR‐30c antagomirs or pre‐miRs. This result suggests that there is no additivity or cooperativity between miR‐30b and miR‐30c to downregulate Runx2 and is consistent with the shared seed sequence within the miR‐30 family. Our findings are also in line with prior studies that identified members of the miR‐30 family (miR‐30a, miR‐30d, miR‐30e) that were downregulated by BMP‐2 or involved in osteogenic differentiation. For example, BMP‐2–downregulated miR‐30a and miR‐30d in murine C2C12 cells and, more recently, miR‐30c was identified as part of a program of miRs that target Runx2 to control osteoblast maturation and are inversely expressed in relation to Runx2 expression.^18–20^ Here, miR‐30c expression in murine MC3T3‐1 premature osteoblasts, ATDC5 chondrocytes, or NIH3T3 fibroblasts was shown to downregulate Runx2 expression to a different degree in each cell type. This finding suggests that the effect of miR‐30c on Runx2 expression may have cell or tissue specificity.^20^ In addition, while we focused on Runx2 as the target of miR‐30b and miR‐30c, it is also possible that other targets of miR‐30b or miR‐30c not examined in our study mediate CASMC calcification.

The mechanism(s) by which miR‐30b and miR‐30c expression is downregulated by BMP‐2 remains unclear. We investigated Smad signaling as one possible mechanism as *Smad1*/5 signaling has been shown to be involved in miR processing, albeit to increase miR maturation by Drosha.^26,33–34^ Our results do not support a role for *Smad1* in the BMP‐2–mediated downregulation of miR‐30b and miR‐30c as transfection of cells with an siRNA to *Smad1* had no effect on miR‐30b or miR‐30c expression in CASMCs. The difference between our findings and prior studies may be explained, in part, by the observation that miR‐30b and miR‐30c were not identified as miRs that contain a conserved or variant regulatory Smad binding element that was present in the miRs regulated by Smad signaling.^26^ It is also possible that miR‐30b and miR‐30c expression is modulated by transcriptional regulation. In a screening study of 175 human miRs, one‐third of intronic miRs were found to have transcription initiation regions independent from their host promoters. In this study, human miR‐30c1 and miR‐30e were identified as miRs that have conserved intronic miRNA clusters using independent intronic promoters.^35^ miR‐30b is an intergenic miR located in 8q24.22 (−135 812 850: −135 812 763) between the protein coding regions for ZFAT and ribosomal protein L23 pseudogene 56 (RPL23AP56). Mature miR‐30c may be produced by 2 transcripts: miR‐30c1 (Gene ID: 407031) in 1p34.2 (41 222 956; 41 223 044), which is intronic and overlaps with nuclear transcription factor Y, γ subunit, and miR‐30c2 (Gene ID: 407032), which is intronic and is located in 6q13 (72 084 663:72 088 734) between the genes for opioid growth factor receptor‐like 1 and RP1‐288M22.2, a processed transcript for which there is no protein product. This suggests that elements that regulate transcription of both the primary transcripts as well as neighboring genes may also regulate miR‐30b or miR‐30c transcription.

In summary, we find that BMP‐2 downregulates miR‐30b and miR‐30c to increase Runx2 expression and promote CASMC calcification. The finding that these miRs regulate acquisition of an osteoblast‐like phenotype in SMCs and other cell types, including preosteoblast cells and chondrocytes, confirms the tenet that vascular calcification recapitulates bone formation. These studies also identify miR‐30b and miR‐30c as potential candidates to modulate ectopic vascular calcification that may be associated with comorbid diseases, such as diabetes mellitus and end‐stage renal disease. Finally, owing to the similarities between osteoblast and SMC miR‐30b‐c and Runx2 expression, additional study is warranted to determine if there are tissue‐specific features or regulators of these miRs to allow for targeted miR‐specific interventions to modulate vascular calcification without influencing endochondral bone formation.
